# [Retracted] Inhibition of δ‑opioid receptors induces brain glioma cell apoptosis through the mitochondrial and protein kinase C pathways

**DOI:** 10.3892/ol.2023.13768

**Published:** 2023-03-20

**Authors:** Lixiang Zhou, Xudong Guo, Mo Chen, Shuanglin Fu, Jingbin Zhou, Gang Ren, Zirong Yang, Wenhai Fan

Oncol Lett 6: 1351-1357, 2013; DOI: 10.3892/ol.2013.1546

Following the publication of this paper, it was drawn to the Editor's attention by a concerned reader that the western blotting data shown in Fig. 3C on p. 1354 for the Bak protein bands were strikingly similar to bands that appeared in another article written by different authors at different research institutes, which had already been submitted for consideration for publication [Li ZH, Yu Y, Du C, Fu H, Wang J and Tian Y: RNA interference-mediated USP22 gene silencing promotes human brain glioma apoptosis and induces cell cycle arrest. Oncol Lett 5: 1290-1294, 2013].

Owing to the fact that the contentious data in the above article had already been submitted for publication elsewhere prior to its submission to *Oncology Letters*, the Editor has decided that this paper should be retracted from the Journal. The authors were asked for an explanation to account for these concerns, but the Editorial Office did not receive a reply. The Editor apologizes to the readership for any inconvenience caused.

